# Analysis of Wilson disease mutations in copper binding domain of *ATP7B* gene

**DOI:** 10.1371/journal.pone.0269833

**Published:** 2022-06-28

**Authors:** Bushra Gul, Sabika Firasat, Raeesa Tehreem, Tayyaba Shan, Kiran Afshan

**Affiliations:** 1 Department of Zoology, Faculty of Biological Sciences, Quaid-i-Azam University, Islamabad, Pakistan; 2 Department of Biosciences, Faculty of Basic Sciences, University of Wah, Wah Cantt., Pakistan; National University of Kaohsiung, TAIWAN

## Abstract

Wilson’s disease (WD) is an autosomal recessive disorder, resulting from variations in *ATP7B* gene. Clinical heterogeneity, including neuropsychiatric and hepatic manifestations over a large range of age groups make diagnosis difficult. Most of WD patients suffer severe disabilities and even die. So, overall goal of proposed study is the genetic and clinical characterization of Wilson’s disease cases from Pakistani population. Clinical data was collected, and patients were investigated for variations in selected *ATP7B* exons using PCR based Sanger sequencing. Pathogenic effect predictions for detected variants were carried out using PROVEAN, MutationTaster2, and HSF software’s. Clinical heterogeneity was observed in patients including reduced serum ceruloplasmin, signs of chronic liver damage and raised 24 h urinary copper excretion. Mean age of onset was 11.3 years. Kayser-Fleischer rings were present in 75% of cases. About 82.5% patients belonged to inbred families. Patients having neurological disorder were above 12 years of age. Total ten variants in analyzed region of *ATP7B* gene, including a reported variation (p. L227Yfs*35) were found in patients. The study also identified 4 putative novel synonymous variants (c.251A>C, c.15T>A, c.6T>C, c.238C>T) and 5 reported polymorphisms (c.83C>A, c.39_40insCGGCG, p.V456L, c.39_40insCGCCG and c.1544-53A>C). Reliable understanding of clinical presentations and genotype-phenotype correlation provide insight to function and structure of *ATP7B* and may assist in disease prognosis and family counseling. The study revealed clinical presentation of Pakistani WD cases and identification of sequence variants in screened region of *ATP7B*.

## Introduction

Wilson’s disease (WD-MIM#277900), an autosomal recessive disease of copper metabolism, results in excessive copper deposition, primarily in the liver and the brain, leading to hepatic and neuropsychiatric manifestations [1]. It is a monogenetic disease with diverse clinical heterogeneity in patients with hepatic, corneal and neurological involvement [2, 3] which makes its clinical diagnosis challenging. Biliary excretion is the only mechanism for excessive copper excretion that is why compromised in copper excretion in WD affected individuals’ leads to progressive copper accumulation in the liver [4]. For WD management, copper chelation through penicillamine is considered as an effective therapy in most of the patients and liver transplantation is curative when patients present irreversible liver failure, but liver transplantation is very expensive. Low copper diet, Zinc supplements and trientine treatment are also strongly recommended [5].

Excessive copper excretion via *ATP7B* dependent mechanism is the major homeostatic pathway for copper metabolism. *ATP7B* gene is located on chromosome 13, comprised of 21 exons that code for a 1465 amino acid protein [5–7]. *ATP7B* protein is expressed in trans-Golgi network of hepatocytes in normal copper state but in case of copper excess it translocates to canalicular plasma membrane to promote copper excretion. Genetic alterations in *ATP7B* may compromise protein function resulting in copper buildup in hepatocytes and brain as well as defective synthesis of ceruloplasmin [8].

To date over 877 homozygous or compound heterozygous variations have been identified in *ATP7B* related to Wilson Disease (WD), which have been documented from various countries (The Human Gene Mutation Database (HGMD®). Available at: http://www.hgmd.cf.ac.uk/ac/index.php, Accessed: 17 May 2020) [5]. Owing to recessive inheritance pattern of WD, its incidence is higher in populations with customary consanguineous marriages like Pakistan, however only few variations have been reported from Pakistan (p.Cys271X, p.Val456L and p.V272V) necessitating comprehensive clinical and genetic studies on this disease from our local population [9–11].

The present study was aimed to show clinical heterogeneity among forty Pakistani WD patients presented at tertiary care hospitals. Our study unveils clinical and genetic heterogeneity of WD in our population. We aimed to investigate the variants in copper binding domain of ATP7B gene that affect the binding of copper and its excretion that is the main cause of disease pathogenesis.

## Materials and methods

### Sample collection

The Bioethical Committee of Quaid-i-Azam University, Islamabad, Pakistan approved this study on 14th October 2017. Patients’ age at time of WD diagnosis was determined. Age was divided into the following categories: Elementary School (5–10 years); High School/Adolescent (11–15 years); (16–20 years), and (21–25 years) (Table 1). Forty WD patients belonging to inbred families along with available asymptomatic family members were recruited through expert physicians from the Neurology and Gastroenterology departments of Pakistan Institute of Medical Sciences (PIMS) and Children Hospital Lahore (CHL) from November 2017 to November 2018. All participants provided written informed consent according to the tenets of the Declaration of Helsinki.

**Table 1:** Demographic information of children with Wilson’s disease in Pakistan.

		Frequency (n = 40)	Percentage (%)
**Sex**			
	Male	20	50
	Female	20	50
**Age at referral**			
	5–10 years	24	60
	11–15 years	9	22.5
	16–20 years	3	7.5
	21–25 years	4	10
PCM		33	82.5
Fam Hist		14	35
KF ring		11	27.5
Jaundice		32	80
Seizures		4	10
Paralysis		2	5
Decreased alertness		13	32.5
Poor cognitive ability		14	35

### Clinical assessment

Clinical record of patients including presence of KF ring, elevated 24-h urinary copper, lowered plasma ceruloplasmin, and raised liver copper concentrations was obtained (Table 2) [1, 12]. Complete family history of disease and consanguinity was recorded for forty enrolled cases. Neurological disabilities including cognitive abilities, decreased alertness and paralysis were recorded along with hepatic manifestations. The age of diagnosis was between 5 to 25 years (Table 1). Five milliliters of peripheral blood were collected from twenty affected cases and their unaffected family members for genetic analysis.

**Table 2:** Clinical profile of enrolled 40 patients with Wilson’s disease.

Patient ID	PCM	Fam Hist	KF ring	Hepatic Disabilities				Neurological Disabilities			
				CPL mg/dl	UI Cu μg/day	TSB	JD	SZR	PAR	Dec. alert.	Poor cogn. Abl.
WD-1	Y	-	Y	0.1	1450	15	Y	-	-	-	-
WD-2	Y	-	Y	0.16	1654	24	Y	-	-	-	-
WD-3	Y	-	-	0.16	690	5	Y	-	-	-	-
WD-4	-	-	-	0.11	1365	1.9	Y	-	-	-	-
WD-5	-	Y	Y	0.14	1254	18.5	-	-	-	Y	Y
WD-6	Y	Y	-	0.12	1432	3.9	Y	-	-	-	-
WD-7	Y	-	Y	0.12	550	4.6	Y	Y	-	Y	Y
WD-8	-	-	-	0.15	1390	2.6	-	-	-	Y	Y
WD-9	-	Y	Y	0.13	770	15	Y	-	-	-	-
WD-10	Y	-	Y	0.12	1230	26	Y	Y	Y	Y	Y
WD-11	Y	Y	Y	0.11	1245	1.6	Y	-	-	-	-
WD-12	Y	Y	-	0.13	987	4.6	Y	-	-	-	-
WD-13	Y	Y	Y	0.03	890	2.9	Y	-	-	-	-
WD-14	Y	Y	Y	0.05	1390	3.8	Y	-	-	-	-
WD-15	-	-	Y	0.06	1136	4.4	Y	-	-	-	-
WD-16	Y	-	Y	16	1438	9	Y	-	-	-	-
WD-17	Y	-	Y	0.11	970	2.9	Y	-	-	Y	Y
WD-18	Y	Y	Y	0.21	1200	3.9	-	Y	-	Y	Y
WD-19	Y	Y	-	0.5	1360	3.3	-	-	-	Y	Y
WD-20	Y	-	-	17	1405	1.8	Y	-	-	-	-
WD-21	Y	-	Y	0.11	1330	2.1	Y	-	-	-	-
WD-22	Y	Y	Y	0.5	1100	3.3	Y	-	-	Y	Y
WD-23	Y	Y	Y	15.5	1300	5	Y	-	-	-	-
WD-24	Y	-	Y	0.25	1450	20	Y	-	-	Y	Y
WD-25	-	Y	Y	0.11	1550	15	Y	-	-	-	-
WD-26	Y	Y	Y	0.05	960	4.6	Y	-	-	-	-
WD-27	Y	-	Y	0.5	1400	3.3	Y	-	-	-	-
WD-28	Y	Y	Y	0.06	1360	3.9	Y	-	-	Y	Y
WD-29	Y	-	Y	0.16	950	20	Y	-	-	-	-
WD-30	Y	Y	Y	0.7	800	1.8	Y	-	-	-	-
WD-31	Y	-	Y	15.5	1300	3.3	-	-	-	-	-
WD-32	Y	-	-	14.5	1244	3.9	Y	-	-	-	-
WD-33	Y	Y	Y	0.13	1305	1.8	Y	Y	Y	Y	Y
WD-34	-	Y	Y	0.13	1400	20	-	-	-	Y	Y
WD-35	Y	-	-	0.1	1350	3.3	Y	-	-	-	-
WD-36	Y	-	Y	0.05	1100	5.8	-	-	-	-	-
WD-37	Y	-	Y	0.09	863	3.7	Y	-	-	-	-
WD-38	Y	Y	Y	0.12	790	20	Y	-	-	-	-
WD-39	Y	-	-	0.06	770	4.8	-	-	-	-	Y
WD-40	Y	-	Y	0.14	1530	4.9	Y	-	-	Y	Y

**Gen:** Gender, **AOD:** Age of diagnosis, **Fam Hist.:** Family History, **KF:** Kayser-Fleischer rings, **CPL:** Ceruloplasmin, **UI:** Urinary, **TSB:** Total serum bilirubin (Normal range: 0.1–1.2 mg/dl) **JD:** Jaundice, **SZR:** Seizure, **PAR:** Paralysis, **Dec. alert.:** Decreased alertness, **Poor cogn. Abl.:** Poor cognitive abilities, **HD**: Hepatic disease, **PCM**: Parental Cousin Marriage, **ND**: Neurological Disorder

### DNA extraction

Genomic DNA was extracted from collected blood samples by phenol-chloroform methods [13]. Quantity and purity of extracted DNA was accessed using a μDrop Plate reader (MultiskanTM, Thermo Fisher Scientific, and Waltham, MA, USA). The extracted DNA was preserved at 4°C before further use.

### Primer designing and PCR

Primers of exon 1–4 of *ATP7B* gene were designed by using Primer3 software (http://bioinfo.ut.ee/primer3-0.4.0/) (Table 3). A 2.5 μl DNA sample of each proband was amplified in 25 μl of PCR reaction containing 0.3 μl of Taq DNA Polymerase (5 U/μL ((Thermo Scientific Inc.), 2.5 μl of 2.5 mM deoxynucleotide. Triphosphate mixture, 0.5 μl of each forward and reverse primer (10 pmol/μl), 2.5 μl of 10 × reaction buffer (without MgCl2) and 2.5 μl of MgCl2 (25 mM). The PCR T100 thermal cycler (Bio-Rad, CA, USA) was used with a cycling program of initial denaturation temperature 95°C for 5 min, followed by 10 cycles of 95°C for 45 sec, 69°C– 64°C (according to melting temperature of each primer pair) for 45 sec with an increment of -1 in each subsequent cycle, 72°C for 45 sec again followed by 30 cycles of 95°C for 45 sec, 59°C– 54°C (according to melting temperature of each primer pair) for 45 sec, 72°C for 45 sec and a final extension at 72°C for 10 min followed by a final hold at 25°C. The amplification PCR products were loaded on the 1.5% agarose gel along with 1 kb size ladder to evaluate product size and purified by using DNA purification Kit (Wiz Bio Solutions, Seongnam, Korea).

**Table 3:** Primer sequences for amplification of 4 selected exons of *ATP7B* gene.

Exon No.	Primer Sequence (5’ → 3’)		Size (bp)	Annealing temp. (°C)
1	F	GCAACTTTGAATCATCCGTGT	326	60
	R	AAAATCCTCCTGGTGGGAGT		
2A	F	TAGATGCTGCCTTTAGCTTGC	766	58
	R	TAAGGGAGCCACTTTGCTCTT		
2B	F	TCTCACTCAGCAACCAAGAGG	847	60
	R	AGGGCTCACCTATACCACCAT		
3	F	CTCACCAAGAGCCCTGAAAC	394	60
	R	CGAGGTCTATACGCAGCATTC		
4	F	GGGTAAGAGACCAGACATCGT	473	58
	R	AACAAACCAGACACGTCCAAG		

### Sanger sequencing and data analysis

The samples were then sequenced by DNA Core Facility, Centre for Applied Molecular Biology, Lahore Pakistan. The sequenced data was analyzed by using Sequencher 5.4.6 software. Pathogenicity prediction for each variant was done by various bioinformatics tools named MutationTaster (http://www.mutationtaster.org/), Mutalyser (https://mutalyzer.nl/), polyphen-2 (http://genetics.bwh.harvard.edu/pph2/), PROVEAN (http://provean.jcvi.org/index.php), Mutation assessor (http://mutationassessor.org/r3/), and SIFT (http://sift.jcvi.org/) and public database frequency was also determined. HSF (Human Splice Site Finder) software version 3.0 (www.umd.be/HSF3/) was used to determine effects of sequence variations on exonic splicing signals [14].

## Results

### Clinical evaluation

**Fig 1:**
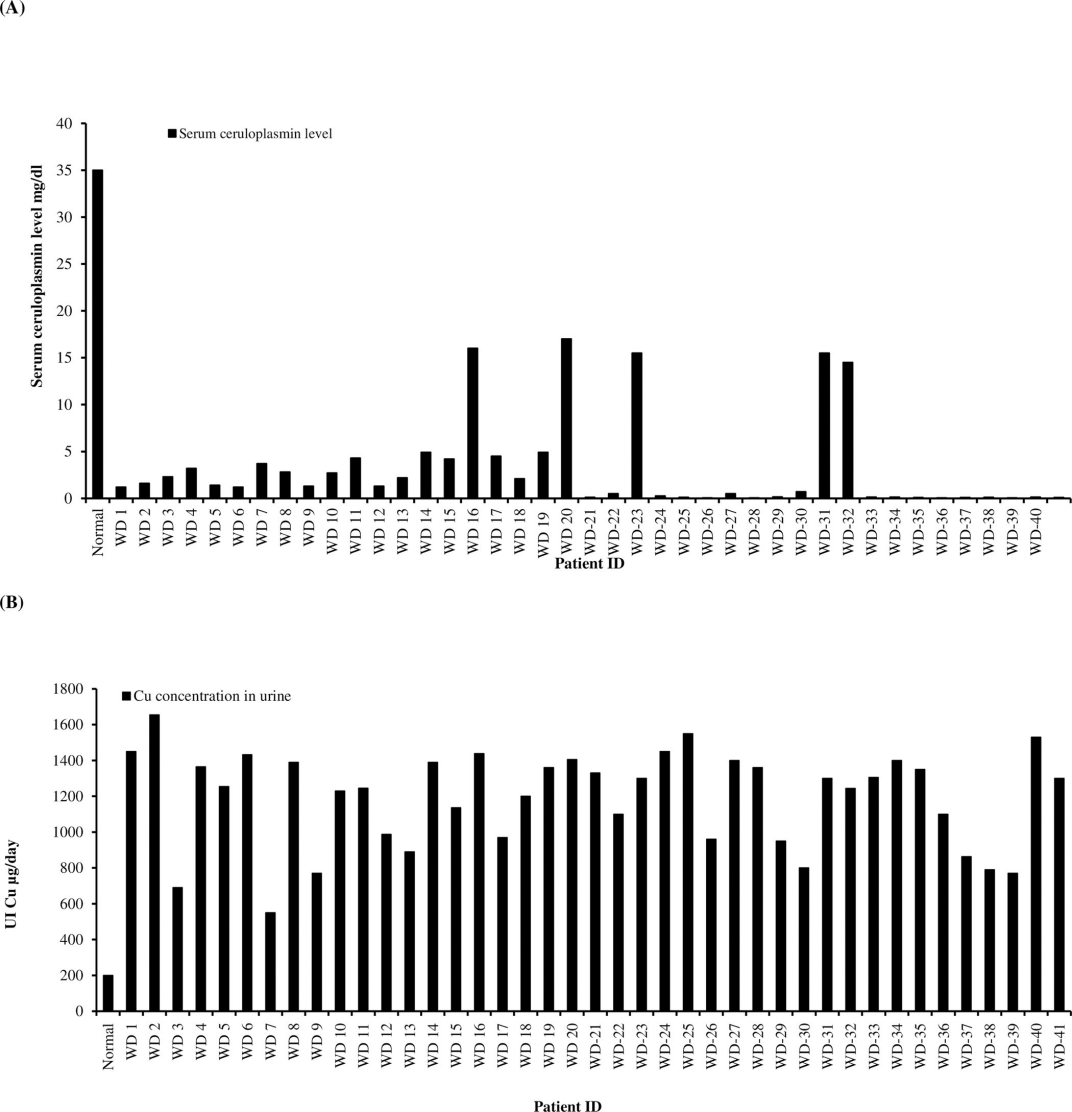
(A) Graph showing decreased serum ceruloplasmin level in all patients suffering from WD as compared to normal serum ceruloplasmin level among patients enrolled in this study and (B) Graph showing increased copper concentration in urine than normal 200μg/d in patients included in this study.

**Fig 2:**
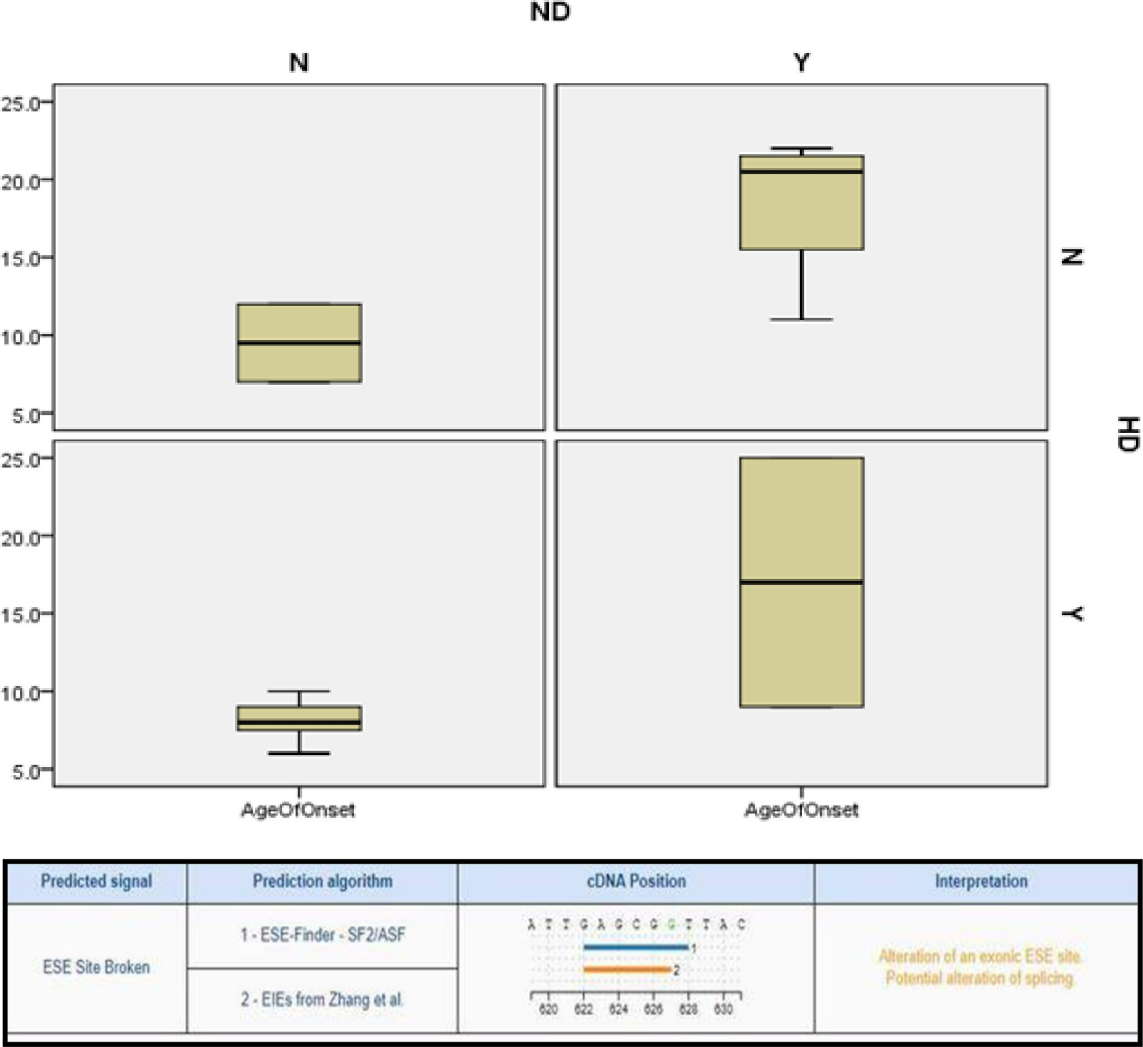
(A) Demonstrating younger age of onset of patients with initial liver disease, compared to patients with neurological manifestations in a Pakistani cohort (B) Pathogenic effect prediction output of HSF program for variation L227Yfs*35).

Based on the generation skip, affected individuals having unaffected parents and patients born to consanguineous parents’ autosomal recessive inheritance of the WD was demonstrated in all enrolled cases. The age of diagnosis of WD in probands of enrolled families was between 5–25 years with male to female ratio of 1:1. Clinical characteristics of each proband are listed in Table 2. WD was detected based on characteristic clinical features including high urinary copper levels, Kayser-Fleischer rings (75% of the cases), high liver copper, and low serum ceruloplasmin and abnormal brain magnetic resonance imaging. Decreased serum ceruloplasmin level and increased copper concentration in urine in all patients suffering from WD enrolled in this study is shown graphically in Fig 1A & 1B. Of the forty cases 24 had only hepatic involvement (62%), six had only neurological (15%) symptoms, two were asymptomatic (5%) and eight had both hepatic and neurological manifestations (20%). The KF ring was detected in thirty patients (75%). 82.5% were products of marriage between first cousins (Table 2). Patients having neurological illness were above 12 years of age, Children below 12 years mostly had hepatic disorders; with the mean age of onset of 11.3 years, signifying that the onset of neurological disabilities are related with disease prognosis and age (Fig 2).

### Genetic screening of selected exons of *ATP7B*

**Fig 3:**
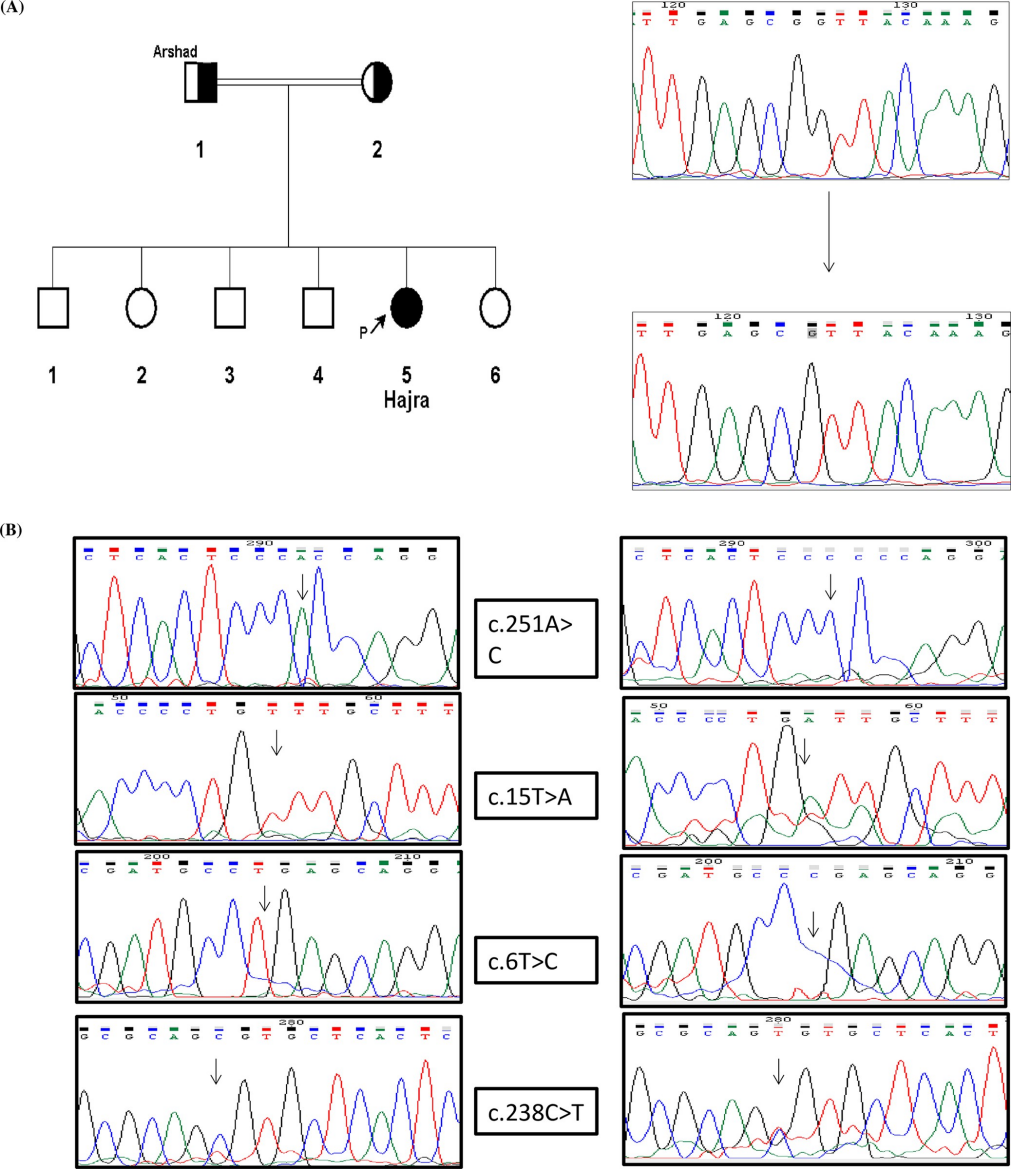
(A) Pedigree of family of Patient WD-1 and Chromatogram of variation found in exon2 of *ATP7B* and (B) Chromatograms for Novel Variants found in *ATP7B* gene.

Sequencing of 4 selected exons of the *ATP7B* gene in 20 out of 40 enrolled patients revealed 10 variants in intronic, exonic and untranslated regions (UTR’s) (Table 4). 40% of these identified putative novel synonymous variants (g.251A>C, cDNA.15T>A, cDNA.163T>C, g.238C>T), 5 known polymorphisms (cDNA.83C>A, cDNA.39_40insCGCCG, cDNA.39_40insCGGCG, p. V456L and g.42835A>C) and one was reported pathogenic variation (p. L227Yfs*35) affecting Cu binding domain of the protein. According to Insilico analysis this variation is listed as Probably Damaging with Polyphen 2 score of 0.995. Human Splicing Finder (HSF) considered the variant as possible source of splicing alteration, through altering an exonic splicing silencer (ESS) site and breaking an exonic splicing enhancer (ESE) (Fig 3).

**Table 4:** Shows variation found in selected exons (1, 2, 3, 4) of *ATP7B* gene along with their frequency among twenty patients (n = 20) included in this study.

Nucleotide Change	Exon/Intron	Amino acid	Insilco prediction	Polyphen 2		Functional Domain	Patient frequency (%)	Reference ID	Novelty
				Prediction	Score				
NM_000053.3:C·83C > A	5’UTR*	-	Polymorphism	**-**	**-**	5’UTR	35	rs2277448	-
NM_000053.3:C.39_40insCGCCG	5’UTR	-	-do-	-	-	5’UTR	10	rs3832920	-
NM_000053.3:C.251A>C	Intron 1	-	-do-	-	-	Cu** Binding	5	-	Novel
NM_000053.3:C.15T>A	5’UTR	-	-do-	-	-	5’UTR	5	-	Novel
NM_000053.3:C.6T>C	Exon 1	-	-do-	-	-	Cu Binding	10	-	Novel
NM_000053.3:C.39_40insCGGCG	5’UTR	-	-do-	-	-	5’UTR	5	-	-
NM_000053.3:C.238C>T	Intron 1	-	-do-	-	-	Cu Binding	5	-	Novel
NM_000053.3:C.678_678delG	Exon-2	L227Yfs*35	Disease Causing	Probably Damaging	0.995	Cu3	5	-	-
NM_000053.3:C.1366G>C	Exon 3	V456L	Polymorphism	Benign	0.001	Cu4/Cu5 Binding	40	rs1801244	-
NM_000053.3:C.1544-53A>C	Intron 3	-	-do-	-	-	Cu Binding	100	rs2147363	-

*un-translated region

**copper

## Discussion

Clinical presentation of Wilson disease (WD) is very heterogeneous with predominant reports of hepatic, neurologic, and ophthalmic involvement along with multiple other abnormalities ranging from hemolytic anemia, thrombocytopenia, renal and gynecological problems as well as bone and muscle issues [15]. Disease may remain undetected initially and in most of the cases it is diagnosed after substantial damage to liver making liver transplantation the only treatment option [15]. Genetic diagnosis provides early disease detection which may help to reduce disease associated morbidity and high mortality especially in high-risk consanguineous populations such as our Pakistani population. Furthermore, to establish genotype-phenotype correlations, clinical and genetic data from different ethnicities is essential. For this purpose, we enrolled forty WD cases presented at two tertiary care hospitals. Among enrolled cases 95% cases belonged to inbred families with 82.5% being products of first cousin unions (Table 2). This observation is attributed to high degree of consanguinity, a main determinant of the high incidence of recessive disorders in our population [16, 17]. Furthermore, 5% cases were not belonging to consanguineous parents which show presence of minor allele in general population with possibility of compound heterozygous variations predisposing to clinical manifestation [18]. Eighteen out of 40 probands had a positive family history of disease (Table 2).

Analysis of clinical records of forty cases revealed that Kayser-Fleischer (KF) ring was present in corneas of 75% of cases at the time of diagnosis (Table 2). According to Rangaraju et al., 2015 KF ring is detected in corneas of 95% of WD cases [15] which is not consistent with our data. Possible explanations for this difference can be nonappearance of detectable KF ring in cases with hepatic manifestation and early staging of disease in children as 7/10 cases without KF ring had only hepatic symptoms whereas 8/10 cases were ≤ 15 years of age (Table 2) [19, 20] Clinical details of cases showed that all forty cases had high urinary copper concentration and low serum ceruloplasmin levels. Among these twenty six (65%) and fourteen (35%) cases had hepatic and hepatic as well as neuronal symptoms at the time of diagnosis, respectively. However, two cases (WD-7 & WD10) that initially had only hepatic involvement later developed neuronal problems as well (Table 2). Box plot shown in Fig 2, demonstrates that patients presenting with only liver disease at the time of diagnosis had younger age of onset as compared to patients with both hepatic and neurological symptoms (Fig 2), this data is consistent with previous report [10]. All patients without neurological symptoms are in their first or second decade of life and the possibility to develop neurological issues later in their lives could not be excluded [21]. Along with these clinical symptoms, sever joint pain, yellowing of skin, recurring fever and abdominal swelling were the common feature in most of the patients.

Variations in the efflux copper transporter *ATP7B* leading to toxic copper accumulation predispose to WD phenotype. Some of *ATP7B* variations appear to be population specific, while others are found in probands from variety of diverse ethnic families [20, 22, 23]. Mutational data from Asian countries is mainly available from China, Japan, South Korea and India [24] but there are only a few reports regarding molecular genetic screening performed on a total of 12WD cases from our Pakistani population [10].

However, to understand disease mechanisms and to establish genotype phenotype correlations extensive genetic studies are warranted especially from populations with expected high prevalence. Wilson’s disease is an autosomal recessive disorder of copper transport involving accumulation of copper in liver and brain of affected individuals. Defective excretion of copper appears to be the most important cause of copper accumulation in Wilson disease. Mutations that impair transport activity or disrupt intracellular targeting of ATP7B cause Wilson disease and chronic copper toxicosis. Therefore, we did molecular analysis of copper binding domain, exon 1–4 of *ATP7B* for twenty enrolled cases based on availability of funds and blood samples. WD cases that had blood transfusion within three months prior to enrolment were not included in genetic analysis. Screening of exon 1–4 of *ATP7B* as well as intron exon boundaries revealed 10 sequence variations including four novel synonymous variants, 5 known polymorphisms and one reported variation, affecting Cu binding domain of the protein (Table 4). The identified variation is a single base deletion i.e., cDNA.835_835delG/c. 678delG1 predicted to cause a shift in reading frame and premature truncation of mutated protein (L227Yfs*35). This disease-causing variant is detected in exon 2 of *ATP7B* in a 7-year-old boy belonging to consanguineous family (WD-1) (Table 2).

This variation was initially identified by Aggarwal in 2013 in a patient from Western Indian population [25]. The resultant truncated protein has reduced ability of biliary excretion of copper and affected individuals may harbor a moderate to severely disabling phenotype with limited variability. According to Insilco analysis variations p. L227Yfs*35 was predicted to be probably damaging with Polyphen 2 score of 0.995. Human Splicing Finder (HSF) analysis revealed that a guanine nucleotide deletion i.e., cDNA.835_835delG may cause splicing alteration through alteration of an exonic splicing silencer (ESS) site and breaking an exonic splicing enhancer (ESE) (Fig 3). Exonic splicing enhancers (ESE) are the discrete sequences within the exons that promote both regulated and constitutive splicing. ESS sequences function to suppress splice site selection and often appear to operate in conjunction with ESE sequences, which, when activated, are dominant over the adjacent ESS sequence. Changes in one or more factors required for normal ESE and ESS functions are responsible for the deregulation of alternative splicing events in pre-mRNA [26].

A synonymous reported polymorphism (chr13:52585548G>T) (rs2277448) was found in homozygous condition in five patients i.e., WD3, WD4, WD5, WD10, WD19 and in heterozygous condition in 2 patients i.e., WD1 and WD15. This variant was found in 5’ Un translated region (UTR) of exon-1 so is not related to protein, but it may activate crypter donor site with potential effects on splicing as predicted by Human Splicing Finder. It might be playing regulatory roles in protein expression regulation and further extensive studies are required to find its association with disease. Two five nucleotide insertions i.e., (cDNA.39_40insCGCCG) and (cDNA.39_40insCGGCG) (rs3832920) upstream of exon 1 in 5’UTR region detected in two patients (WD4, WD10) and a patient WD11 respectively were also predicted to create an ESS site and breaking an ESE by HSF analysis. A known homozygous variant in exon 3, p. V456L (rs1801244), also previously reported from Pakistan and India might be having a polymorphism found in 40% cases with amino acid change V456L, is also a potential reason of affecting the splice site [27].

Two other novel variants c.163T>C and c.15T>A in 5’UTR of exon 1 were detected in heterozygous condition each in four and two patients respectively were predicted by Human splicing finder to affect splicing of transcript (Table 4; Fig 3). Additional pathogenic variants are expected to locate in the unsequenced exons of ATP7B or any other gene. As we did not screen all exons of *ATP7B* due to financial limitations therefore we could not provide genetic diagnosis of our enrolled cases. Further analysis of remaining exons is required which will not only reveal homozygous or compound heterozygous disease-causing variants in all these patients, but the Insilco analysis of all identified variants will help to establish a possible genotype-phenotype correlation. The predicted polymorphic variants which might affect splicing can aggravate outcomes of same variation in different patients resulting in phenotypic variability of disease.

The mutational pattern in *ATP7B* gene is highly nonoverlapping in different regions of the world. The most common variation found in mixed European populations is H1069Q which is responsible for 35–45% of WD alleles in these populations [28]. Another common variation i.e., R778L is prevalent in Asian populations where it accounts for more than 20% of all WD alleles [29].

High suspected incidence of disease due to consanguinity, scarcity of molecular genetics data, clinical heterogeneity of WD with challenging disease diagnosis and high mortality and economic burden of end stage disease treatment option i.e., liver transplant necessitates extensive genetic studies on this disease in local population. Such studies will help in early accurate genetic diagnosis of affected cases and their asymptomatic family members for regular follow-ups aiding to patient management and genetic counseling. Furthermore, the data generated through these studies will provide genotype-phenotype correlation of local WD patients, improve the awareness regarding clinical presentation of WD to our health care providers hence reducing chances of late diagnosis linked morbidity and mortality in upcoming future.

## Supporting information

S1 Data (XLSX)
